# Beyond QTL and GWAS: how deep learning, graph models, and multi-omics are reshaping plant genomic prediction analysis

**DOI:** 10.3389/fgene.2026.1783939

**Published:** 2026-04-17

**Authors:** Tong Wang, Ran Tong, Zheng Yuan, Yue Li

**Affiliations:** 1 Department of Plant Science and Landscape Architecture, University of Connecticut, Storrs, CT, United States; 2 Department of Mathematical Sciences, University of Texas at Dallas, Richardson, TX, United States; 3 Institute of Chinese Materia Medica, China Academy of Chinese Medical Sciences, Beijing, China; 4 Purdue University, West Lafayette, IN, United States

**Keywords:** artificial intelligence, crop improvement, genomic prediction, genotype-environment interaction, multi-omics integration, nonlinear modeling

## Abstract

**Background:**

Traditional genetic mapping has advanced plant trait studies but struggles to capture epistasis, pleiotropy, and genotype-environment (G × E) interactions in genomic prediction (GP). Recently, artificial intelligence (AI) has provided innovative methods.

**Main body:**

This review outlines the transition from traditional frameworks to AI-enabled approaches for plant trait analysis. Specifically, major statistical and AI methods are summarized; current strategies for combining genomic, transcriptomic, metabolomic, phenotypic, and environmental data are described; and examinations are carried out over how graph-based and Transformer models represent regulatory networks and higher-order interactions. This paper further explores developments in multi-task learning, cross-population and cross-species transfer, and emerging foundation-style models. Key issues related to interpretability, reproducibility, data quality, and evaluation practices are considered in the context of practical deployment.

**Conclusion:**

AI-driven models are reshaping plant trait analysis by extending traditional association methods toward scalable, biologically informed prediction. Continued efforts in data standardization, transparent models, and validation across time and environments will determine the broader impact of these approaches in crop improvement.

## 1 Introduction

Traditional genetic mapping approaches, such as Quantitative Trait Locus (QTL) mapping and Genome-Wide Association Studies (GWAS), have been widely used to identify loci associated with agronomic traits. QTL mapping is typically conducted in bi-parental populations and detects major-effect loci through linkage analysis. In contrast, GWAS evaluates marker-trait associations at the population level to identify significant single nucleotide polymorphisms (SNPs) (Khan et al., 2021). Although these approaches have successfully identified many trait-associated loci, they generally explain only a limited proportion of phenotypic variance because complex traits are often controlled by many loci with small effects.

GP means moving from finding specific loci to predicting genetic importance throughout the whole genome. GP does not look at each marker separately. Instead, it uses data from all genome-wide markers at the same time to figure out a person’s entire genetic potential. With molecular markers distributed across the genome, the overall genetic value can be reliably estimated, despite the limited contribution of each individual marker. These led to Bayesian regression methods, known as the “Bayesian alphabet.” These approaches are still widely used for GP (Meuwiss et al., 2001). GP has mostly used linear mixed models like Genomic Best Linear Unbiased Prediction (GBLUP) and Bayesian alphabet models (BayesA, BayesB, and BayesCπ) to figure out how well a phenotype will perform based on genome-wide marker information (Habier et al., 2007). These models are grounded in quantitative genetic theory, which assumes that phenotypic variation results from the cumulative effects of many loci with small contributions. While linear models are robust and interpretable, they often struggle to capture complex biological mechanisms such as epistasis, dominance, pleiotropy, and genotype-by-environment (G × E) interactions (de los Campos et al., 2013).

In plant breeding, GP is primarily a decision-support framework aimed at guiding selection rather than identifying individual causal loci. Unlike GWAS or QTL mapping, which focus on discovering statistically significant marker–trait associations, GP seeks to estimate genome-wide breeding values to enable early-generation selection, reduce phenotyping costs, and accelerate genetic gain across breeding cycles. Its effectiveness therefore depends on reliable prediction across different years, environments, and populations. In practical breeding programs, where phenotyping resources are limited and environmental variation is substantial, GP supports not only selection decisions but also experimental design and resource allocation within breeding pipelines.

Recent advances in AI have provided new opportunities to address the limitations of traditional GP models. ML and DL approaches can capture nonlinear relationships and complex interactions among genetic markers. In addition, AI-based models facilitate the integration of diverse data types, including genomic, transcriptomic, proteomic, metabolomic, environmental, and phenomic data. These capabilities have raised several key questions for modern breeding research: Can we transfer predictive knowledge across populations, environments, or even species?

- How can these AI models be understood, copied, and used in real breeding pipelines?

**FIGURE 1:**
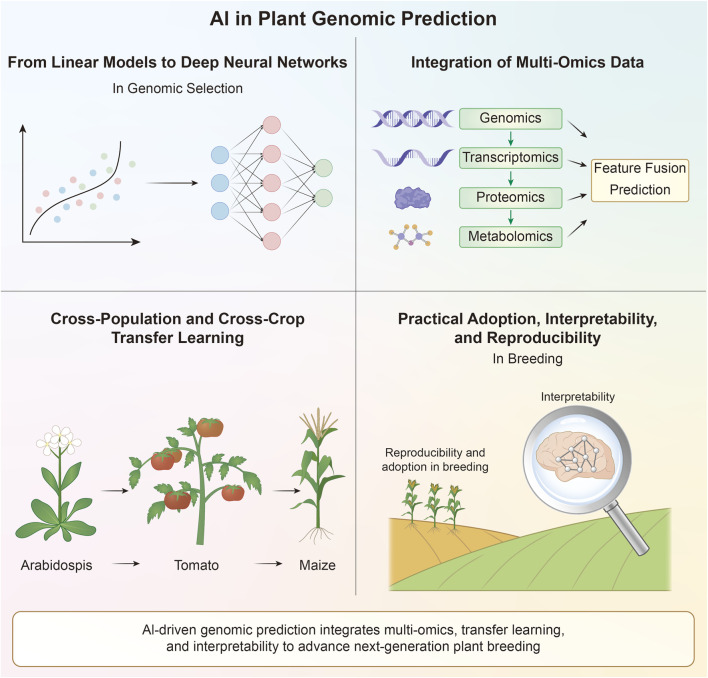
AI-driven plant genomic prediction. (1) AI architectures expand traditional genomic prediction modeling to nonlinear genetic relationships. (2) Multi-omics integration incorporates genomic, transcriptomic, proteomic, and metabolomic data for enhanced prediction. (3) Cross-population and cross-crop transfer learning enables model adaptation from model species to all crops. (4) Practical considerations include interpretability, reproducibility, and computational adoption in breeding, supporting reliable use of AI models in breeding programs.

To address these questions, this review examines recent developments at the intersection of AI and plant genomic prediction. We focus on four major themes: The transition from linear statistical models to AI-based approaches, the integration of multi-omics data, cross-population and cross-crop transfer learning, and the practical challenges associated with deploying AI models in breeding pipelines. By synthesizing recent methodological advances, we aim to clarify how AI is reshaping genomic prediction and how these developments may enhance decision-making in plant breeding (Figure 1).

### 1.1 Scope and review methodology

This review provides a narrative synthesis of methodological advances in plant trait analysis, focusing on the progression from QTL mapping and GWAS to recent AI-enabled approaches. The scope centers on conceptual and algorithmic developments. Literature discussed in this review was identified through non-systematic searches of peer-reviewed articles published over the past two decades, with emphasis on studies introducing new analytical frameworks, multi-omics integration strategies, or cross-population modeling. The objective is to summarize major trends, clarify methodological transitions, and highlight opportunities and constraints relevant to future research and breeding applications.

## 2 Genomic prediction models: from linear methods to artificial intelligence

### 2.1 Traditional linear models for genomic prediction

Against this evolving data landscape, a diverse set of modeling approaches ranging from linear mixed models and conventional ML to DL architectures have been proposed to leverage different data regimes and biological assumptions. Since many agronomic traits are controlled by small-effect loci. In GWAS and genomic selection (GS), statistical models use genomic markers in linkage disequilibrium with these loci to predict trait variation.

A seminal study demonstrates that by constructing and inverting a dense genomic relationship matrix, GBLUP becomes computationally feasible for large-scale breeding populations (VanRaden, 2008). The initial success of GWAS and GS in crops such as maize, wheat, and rice relies on linear mixed models like Ridge-Regression Best Linear Unbiased Prediction (RR-BLUP) and Bayesian “alphabet” models. They treat GP as the estimation of marker effects under specific prior assumptions (Rice and Lipka, 2019). These models are effective when trait variation is primarily driven by many small genetic effects. However, real biological systems are more complex. Gene-gene interactions, pleiotropy, and environmental factors introduce nonlinear relationships between genotype and phenotype. Linear models are unable to fully capture these complexities. For instance, a study compared two genetic models: one based on the parents and the other based on progeny gametic phase (genetic material combinations). The study found that progeny-based models provided a better fit and more accurate predictions. However, when estimating certain genetic effects, such as Additive × Dominance (AD) and Dominance × Dominance (DD), the standard errors were high. In addition, the Additive × Additive (AA) and AD effects could not be clearly distinguished (Bouvet et al., 2016). To overcome the linearity assumption of early GP models, kernel-based regression frameworks were further proposed to capture complex, nonlinear relationships among markers. GBLUP represents a special case of the broader Bayesian framework, while extensions such as BayesA, BayesB, and BayesCπ allow heterogeneous variances and sparse priors on marker effects. The reproducing kernel Hilbert space (RKHS) framework established a theoretical connection between linear mixed models and nonparametric learning, facilitating the flexible modeling of epistatic and regulatory interactions within the genome (Gianola and van Kaam, 2008). The Bayesian Generalized Linear Regression (BGLR) package combines different Bayesian regression approaches, such as shrinkage, variable selection, and semi-parametric methods, into a single Bayesian inference engine. This package provides efficient implementations of models such as BayesA, BayesB, Bayesian LASSO, and Bayesian RKHS regression. Meanwhile, it also addresses the common challenge of high-dimensional data (characterized by a feature count vastly exceeding sample size) in GP (Pérez and de los Campos, 2014).

Moreover, DL offers a flexible data-driven framework to learn such nonlinearities without requiring explicit specification of interaction terms. Early explorations (2015–2018) applying multilayer Perceptrons and Convolutional Neural Networks (CNNs) to genomic datasets yielded mixed results. In 2018, Abelardo Montesinos-López et al. conducted comparative analyses across nine real datasets. Their findings show that DL networks outperform GBLUP without G × E interaction modeling, yet GBLUP is superior when these interactions are explicitly included. These results suggest that DL complements rather than replaces linear models in GS (Montesinos-López et al., 2018).

However, the limited size of breeding datasets at that time often led to overfitting and inconsistent performance. Nevertheless, as larger datasets became available and regularization strategies (e.g., dropout, early stopping, batch normalization) matured around 2020, the potential of DL became more evident (Tian and Zhang, 2022). Montesinos-López and colleagues systematically compared deep multilayer and convolutional architectures across multiple crop datasets, finding that these models performed comparably to or slightly better than GBLUP, particularly for traits with pronounced non-additive genetic components (Montesinos-López et al., 2025). Although the initial performance gains were modest, these studies established DL as a credible alternative to linear methods and sparked the question: is deeper always better?

To provide a structured overview of existing modeling strategies in plant genomic prediction, Table 1 summarizes representative ML studies.

**TABLE 1:** Representative statistical and ML approaches for GP in plants.

Category (statistical)	Model/Method	Task	Data and species	Framework	Interpretability/Validation	References
Statistical linear/mixed models	GBLUP	GS: prediction of quantitative traits and breeding values; stability across multiple environments	SNP genotypes (maize/wheat/rice, etc.)	Linear mixed model with a genomic relationship matrix	High interpretability of marker effects; commonly evaluated using prospective validation (trained up to year N and tested on year N+1)	Montesinos-López et al. (2025), Nishio and Satoh (2015)
Statistical linear models	RR-BLUP	Baseline model for GS	SNP genotypes across multiple crops	Ridge regression linear model	High interpretability; mature and widely used implementation (R package rrBLUP)	Rice and Lipka (2019), Zhang et al. (2010)
Bayesian regression	BayesA/B/Cπ	Estimation of marker effects using different priors (sparse/heterogeneous variance)	SNP data across multiple crops	Bayesian linear regression with different priors	High interpretability; explicit estimation of marker effects	Meuwiss et al. (2001), Habier et al. (2007)
Semi-parametric/kernel methods	RKHS	Capturing non-linear effects and phenotype interactions	SNP data across multiple crops	RKHS kernel regression/semi-parametric model	Capable of capturing non-additive effects; sensitive to kernel choice	Gianola and van Kaam (2008), Sun et al. (2012)
Bayesian toolkits	BGLR	Handling high-dimensional data and integrating multiple models	SNPs, metabolomics, and other omics; multiple crops	Integration of BayesA/B/LASSO/RKHS models	Good interpretability, with both reproducibility and flexibility	Pérez and de los Campos (2014)
Conventional ML regression	RF	Strong baseline performance in medium-sized samples and non-linear scenarios	SNPs, transcriptomics, etc.,; maize and related crops	Decision tree ensemble (bagging)	Interpretable variable importance; strong baseline performance under “large p, small n” settings	Chen et al. (2012)
GWAS-related ML frameworks	ML-GWAS	Identification of G × E-related environment-specific markers and their use in prediction	Soybean scenario: integration of GBLUP and ML-GWAS	ML	Accurate prediction achievable even with very small sets of key SNPs	Verbrigghe et al. (2025)
Shallow multi-omics methods	MGBLUP	Multi-omics integration for predicting breeding values of malting quality	Barley: genomics (SNPs) plus metabolomics (NMR, 24,018 features)	Linear multi-omics integration (structured model)	Strong interpretability of metabolomic features; generalization across populations and environments requires further validation	Guo et al. (2023)

### 2.2 Deep neural approaches for genomic prediction

A significant milestone was achieved with the advent of Deep Neural Network Genomic Prediction (DNNGP), a DL architecture specifically tailored for plant breeding purposes. DNNGP combines different omics layers, such as SNP markers, gene expression patterns, and environmental variables, into a single neural architecture. In benchmarking experiments across four crop datasets (maize, wheat, tomato, and others), DNNGP outperformed five representative baselines, including the traditional GBLUP, two ML models (LightGBM and Support Vector Regression (SVR)), and two earlier DL models (DeepGS and DL-GWAS) (Wang K. et al., 2023; Ma et al., 2018).

DNNGP is well-suited for routine application, as modern major breeding programs encompass tens to hundreds of thousands of individuals with both genotypic and phenotypic data. SoyDNGP represents a CNN-based model for soybean trait prediction, outperforming DNNGP and DeepGS in accuracy and efficiency. It performs well with many crops and features a web server easy to use for trait prediction and genomic analysis (Gao et al., 2023). Parallel Neural Network for Genomic Selection (PNNGS) is a DL model that looks at the data through filters of different sizes at the same time, allowing it to capture both fine details and broader patterns. It works better than classic models such as RRBLUP, Random Forest (RF), SVR, and DNNGP, increasing prediction accuracy by 0.031 over DNNGP. The stratified sampling used in this model helps improve its stability and accuracy, especially when the sample sizes among groups are unbalanced (Xie et al., 2024).

### 2.3 Evolving architectures: from convolution to graph-informed deep networks

Graph Neural Networks (GNNs) and other new neural approaches contribute to interpreting genetic data better compared to traditional networks. As a more advanced technique, these networks use graph topologies to explain how genomic markers are connected in complex ways. Graph-based networks directly depict marker dependencies, facilitating to identify gene-gene interactions and non-linear effects, in contrast to simple feedforward models. It works where genomic data typically display complex patterns that other methods have trouble picking up.

Hybrid architectures that merge CNNs with Recurrent Neural Networks (RNNs), especially Long Short-Term Memory (LSTM) units, have been created to concurrently extract both local and global dependencies from genomic data. The WheatGP model combines CNN and LSTM modules to better forecast the genomes of wheat by considering both short- and long-range genetic connections. It outperforms rrBLUP, XGBoost, SVR, and DNNGP, achieving up to 0.73 accuracy for yield and 0.62–0.78 for other traits. With SHapley Additive exPlanations (SHAP)-based interpretability, WheatGP provides biologically meaningful insights and supports more efficient wheat breeding (Wang C. et al., 2025).

A study introduced two graph-based DL models, a global Graph Convolutional Network (GCN) and a local sub-sampling architecture named GCN-RS, designed for GP using relationship information among individuals. By capturing non-Euclidean graph structures of genomic data, GCN-RS achieved 4.4%–49.4% higher prediction accuracy than GBLUP across simulated and real datasets (wheat, mice, and pig), demonstrating both high efficiency and strong potential for large-scale GP in plants and animals (Kihlman et al., 2024).

Hypergraph Attention Network for Crop Genomic Selection (HGATGS) are established to describe complicated relationships between people instead of just using image-based characteristics to get over the independent-sample assumption in regular GS. In this approach, samples are depicted as nodes interconnected by dynamic hyperedges that signify genetic similarity. Through attention processes and residual connections, the model better captures higher-order interactions and gains improved stability. In assessments utilizing wheat, rice, and maize datasets, HGATGS achieved an accuracy enhancement of up to 66.7% compared to conventional methods, highlighting the potential of graph-structured learning to improve image-based GP in plants (He et al., 2025).

### 2.4 Attention-based models: transformer innovations in genomic prediction

As the Transformer-based models can identify complex, long-range interactions among genetic markers, the emergence of attention-based models, particularly Transformer architectures, has enhanced the capacity to model plant genetic data.

By applying embedding and Transformer-based methods (first developed for language modeling), SNP2Vec frames SNPs in their genomic context. This approach uncovers gene-gene and epistatic interactions that traditional GWAS often overlooks, by capturing the intricate relatedness of genetic variants (Cahyawijaya et al., 2022).

The Genotype-Phenotype Transformer (GPTransformer) analyzes SNPs as sequential data to identify relationships among genetic markers. Using Hardy-Weinberg allele frequencies instead of one-hot encoding improves prediction accuracy by 7%–10%. This representation also helps the model capture both short- and long-range associations between SNPs (Jubair et al., 2021).

The Elastic BERT Model for Genomic Prediction (EBMGP) combines Elastic Net-based feature selection with a bidirectional Transformer encoder and multi-head attention pooling. In this framework, SNPs are encoded as “words,” and groups of SNPs linked by linkage disequilibrium are treated as “sentences.” Across 4 plant and animal datasets, EBMGP outperforms existing approaches in 13 of 16 prediction tasks, with accuracy improvements of up to 9.55% (Ji et al., 2025).

Recent Transformer-based frameworks have been created to capture complicated genetic architecture and make more accurate predictions. These frameworks go beyond existing models. DeepPlantCRE uses both Transformer and convolutional modules to guess how genes will be expressed and find cis-regulatory elements in plants. It performs consistently across different datasets, improving both accuracy and generalizability. It can also pinpoint transcription factor binding sites with good reliability (Wu et al., 2025). The Genotype-to-Phenotype Transformer (G2PT) effectively models nonlinear and epistatic genetic interactions, achieving enhanced predictive accuracy relative to conventional techniques. In the case study of the Triglyceride-to-High-Density-Lipoprotein (TG/HDL) ratio, the proposed model outperformed existing models reported in prior work (Lee et al., 2025).

**FIGURE 2:**
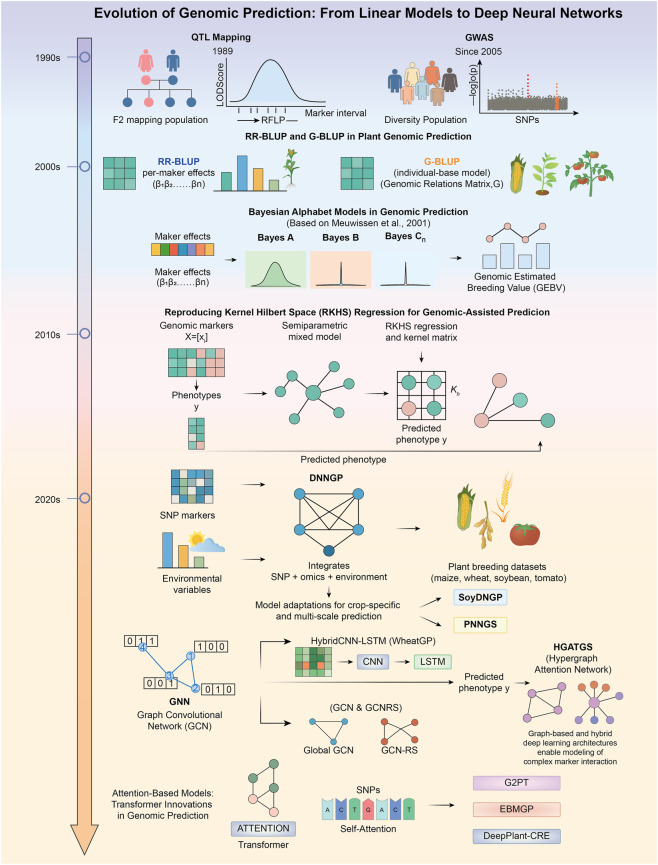
Evolution of Genomic Prediction Models. Genomic prediction has progressed from early QTL mapping, GWAS, and RR-BLUP/G-BLUP to Bayesian regression approaches, having forged the foundation for whole-genome estimation. Kernel and other semi-parametric methods have been introduced to better accommodate nonlinear genetic effects. Recent advances include deep neural networks that integrate genomic, multi-omics, and environmental information, alongside graph- and attention-based models capable of representing genetic structure and long-range regulatory signals.

Collectively, these studies confirm the importance of capturing non-linear relationships for understanding the complexity of genetic variation (Figure 2; Table 2). Newer modeling approaches can make better use of plant genomic data by overcoming the limitations of traditional linear methods.

**TABLE 2:** Representative AI and statistical models used for GP in plants.

Model	Core architecture	Data modalities	Example crops	References
BayesA	Bayesian linear regression	Genomic (SNP)	Livestock, later on plants	Meuwiss et al. (2001)
GBLUP	Linear mixed model	Genomic (SNP)	Livestock, *Maize*, *Wheat*, and *Rice*	VanRaden (2008)
RKHS	Kernel regression/Semi-parametric	Genomic (SNP)	Various species	Gianola and van Kaam (2008)
DeepGS	CNN	Genomic (SNP)	*Wheat*, *Maize*	Ma et al. (2018)
DNNGP	Multi-layer dense neural network	Genomic (SNP), transcriptomics, proteomics	*Maize*, *Wheat*, *Tomato*	Wang et al. (2023a)
HGATGS	Hypergraph Attention Network	Genomic (SNP) + Image-based phenotypes	*Wheat*, *Rice*, *Maize*	He et al. (2025)
EBMGP	Transformer-based hybrid model (BERT)	Genomic (SNP)	*Arabidopsis thaliana*, crops	Ji et al. (2025)
G2PT	Hierarchical Transformer Architecture (Genotype-to-Phenotype)	Genomic (SNP, variants)	Human and transferable to plants	Lee et al. (2025)

## 3 Integration of multi-omics data in prediction model

### 3.1 Multi-omics layers into genomic prediction strategies

The recent adoption of AI in plant GP has been fundamentally driven by the rapid expansion of data resources, including high-density genotyping, transcriptomics, metabolomics, high-throughput phenotyping, and environmental measurements. Compared with early GP studies that relied primarily on SNP markers, contemporary breeding datasets are increasingly high-dimensional, heterogeneous, and collected across multiple environments and time points. These characteristics pose substantial challenges for conventional linear models, but simultaneously create opportunities for models capable of learning complex, non-linear, and cross-modal representations.

Over the past few years, omics technologies have come a long way. These include transcriptomics, proteomics, metabolomics, and epigenomics. Breeders and geneticists have been gradually aware of how these technologies could help them make better predictions about phenotypes, especially for features affected by several things, such as gene regulation, metabolic processes, and reactions to the environment. However, combining these different types of data is quite challenging, as they have multiple dimensions, different scales, and noise that is built in. AI models, particularly DL frameworks, are well-suited to extract predictive patterns from such complex, multi-layered datasets. Over the past decade, there has been a clear shift from relying solely on DNA markers toward building multi-omics predictive models that combine genomics with other features. Multi-omics integration seeks to integrate complementary information from different omic layers and has been broadly classified into five strategies, including early, mixed, intermediate, late, and hierarchical integration (Picard et al., 2021).

In a recent investigation, a multimodal framework was developed. This framework combines genotypic and phenotypic data from the CIMMYT database with high-resolution environmental data from Agricultural ECMWF Reanalysis v5 (AgERA5), effectively improving GP in wheat. The model jointly captures genotype-phenotype-environment interactions and the dynamic effects of environmental variation on gene expression. Using Bidirectional Long Short-Term Memory (Bi-LSTM) and Transformer architectures, it integrates genomic and environmental time-series data and shows strong generalization across different genotypes and environments (Zou et al., 2025).

The studies by Azodi et al. and Riedelsheimer et al. provide compelling evidence for the value of integrating multi-omics data in GP models. Azodi et al. using ML (Bayesian LASSO, RF, and Ensemble) found that integrating transcriptomic data with genomic information markedly improved the accuracy of predicting complex traits in *maize*, such as flowering time. Compared with models based solely on genotypic data, those incorporating transcript levels identified more key genes, providing additional biological insights of transcriptomic information and furnishing a deeper understanding of trait formation mechanisms (Azodi et al., 2020). Similarly, Riedelsheimer et al. using supervised ML RR-BLUP integrated genomic (56,110 SNPs) and metabolomic (130 metabolites) data from *maize* hybrids to predict biomass- and bioenergy-related traits, achieving high prediction accuracies (0.60–0.81) and demonstrating the potential of combined omics approaches for identifying superior hybrid lines (Riedelsheimer et al., 2012). Beyond maize, metabolomic-genomic integration has also proven effective in other crops. Specifically, in barley, Guo et al. used shallow ML combined genome-wide SNPs with 24,018 NMR-based metabolite features for the prediction of malting-quality traits. Their mixed genomic-metabolomic model MGBLUP achieved higher prediction accuracy compared to genomic-only models, illustrating the cross-species potential of metabolomic data in GP (Guo et al., 2023). Gene Network Prediction model (NetGP) serves as a DL framework that integrates genomic and transcriptomic data for phenotypic prediction. By incorporating a novel SNP feature extraction method based on Pearson-Collinearity Selection (PCS) and a gene network architecture, NetGP effectively models cross-omic relationships. The multi-omics version of NetGP performs better than both genomic-only and transcriptomic-only models across a number of plant datasets (Zhao et al., 2025).

### 3.2 Graph-based multi-omics modeling and environmental integration

#### 3.2.1 Graph-based multi-omics architectures

Multi-omics integration has evolved from simple feature fusion to graph-based frameworks that explicitly incorporate biological knowledge. In these models, nodes represent biological entities such as genes or metabolites, while edges represent regulatory or metabolic relationships. Graph Neural Networks (GNNs) propagate information along these edges, enabling the model to capture dependencies among molecular components and trace how genomic variation influences downstream phenotypes. Compared with earlier feature-fusion approaches, graph models better preserve biological structure and can represent complex regulatory interactions within molecular networks (Valous et al., 2024; Leng et al., 2022). In practice, these edges may come from curated pathway/interaction resources, co-expression or statistical association networks, or model-learned dependencies, and thus should not be interpreted as direct reconstructions of experimentally validated gene regulatory networks.

Cross-Omics Graph Convolutional Network (COGCN) works as a graph CNN-based framework for predicting multi-omics phenotypes in *maize*. Rather than simply merging omics inputs, COGCN learns omics-specific features and their nonlinear interactions via a cross-omics tensor framework. By capturing higher-order relationships between genomic, transcriptomic, and metabolomic data, COGCN significantly improves the prediction accuracy of multiple *maize* traits compared to existing methods (Li et al., 2025). Likewise, a bipartite GNN developed to predict maize yield demonstrates that graph-based architectures can also capture cross-sample and trait-environment correlations, enabling both missing-data imputation and accurate yield forecasting even under incomplete or unbalanced conditions (Wang et al., 2024).

Recent progress in image-informed GP uses Unmanned Aerial Vehicle (UAV) phenotyping to link genetic variation with field traits. For instance, a multi-modal DL model was once proposed. This model integrates UAV-derived phenotypic features with genomic variants for yield prediction, achieving a 34.8% accuracy improvement over genotype-only baselines, confirming the potential of image-informed GP frameworks for modern breeding (Togninalli et al., 2023).

Together, these studies underscore the growing utility of graph-structured learning for representing biological and environmental dependencies in plant multi-omics and phenotyping applications.

#### 3.2.2 Environmental data integration and transfer learning extensions

In parallel, environmental data (“enviromics”) has become a critical dimension of predictive modeling. Breeding programs now recognize the need to model G × E interactions by integrating environmental descriptors such as temperature, rainfall, and soil features alongside genomic data. Neural networks that concatenate climatic principal components with SNP markers have shown improved yield prediction stability and enabled the estimation of environment-specific breeding values.

The Transfer-learning Genotype-to-Phenotype framework (TrG2P) approach uses transfer learning on GS by pre-training CNNs on variables that do not affect yield (such plant height and maturity) and then moving the learnt parameters to yield prediction. When tested on datasets of maize, rice, and wheat, TrG2P enhanced prediction accuracy by about 40%, 7%, and 2%, respectively, compared to the best GS models. This shows that using correlated multi-trait information can improve GP performance (Li et al., 2024a).

In soybeans, adding G × E interactions to GP models made them more accurate when conditions changed. The work utilized a combination of GBLUP and ML-based GWAS (ML-GWAS) to identify both general and environment-specific markers, demonstrating that even compact models employing essential SNPs can attain significant predictive power across many contexts (Verbrigghe et al., 2025). A study showed that adding environmental factors like climate and soil data to ML-based GP models can make predictions about maize output up to 7% more accurate than traditional models. The combination of genetic and environmental features (G + E) was more effective and adaptable than explicit G × E interaction modeling. This underscores the importance of envirotyping in enhancing the precision of genetic prediction across various circumstances (Fernandes et al., 2024).

### 3.3 Hybrid prediction and multi-omics synergy

In crops like maize, it is hard to estimate how well a hybrid will do due to dominance and epistasis effect. Using transcriptomic or metabolomic data from parental lines allows models to detect complementation, where one parent compensates for reduced expression of important genes in the other. DL models that combine parental gene expression have been able to make more accurate predictions about hybrid yield. This is because more biological information is added thanks to these omic layers.

The Metabolic Marker-Assisted Genomic Prediction (MM-GP) method improves hybrid breeding by adding important metabolites found in metabolome-wide association studies to GP models. When used on maize and rice, MM-GP enhanced the accuracy of hybrid yield predictions by 4%–14% compared to typical genomic models and was as good as or better than integrated genomic-metabolomic frameworks. This highlights the potential of metabolomic feature selection to improve both accuracy and efficiency in modern hybrid crop breeding (Xu et al., 2025). Furthermore, introducing parental phenotypic data into multi-omics predictive models has been demonstrated to markedly improve hybrid performance forecasting in rice. When genomic, transcriptomic, metabolomic, and phenotypic data were combined, the prediction accuracy improved by 8%–55% across several yield-related characteristics compared to single-omic models. This technique uncovers that adding parental phenotypic features can enhance multi-omics data, giving a better base for hybrid breeding and selection in crops (Xu et al., 2021).

**FIGURE 3:**
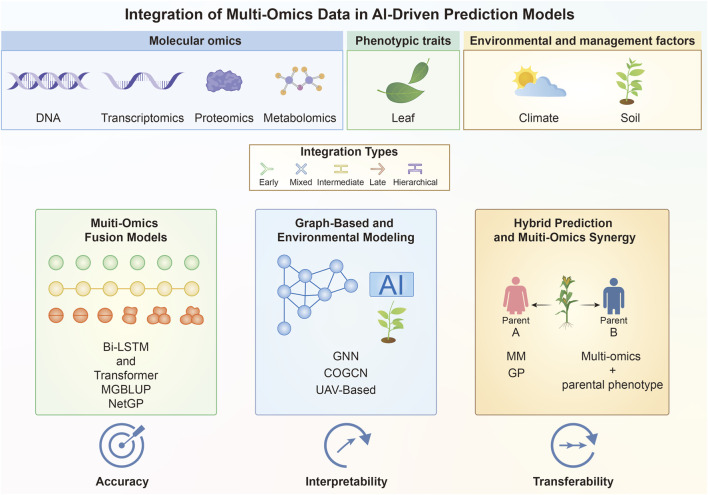
Integration of multi-omics information in data-driven genomic prediction. Genomic, transcriptomic, proteomic, metabolomic, environmental, and phenotype datasets provide complementary views of plant biological variation and can be combined through early, mixed, intermediate, late, or hierarchical integration strategies. Multi-omics fusion models employ sequential and parallel representations to improve predictive accuracy. Graph-based and environment-aware frameworks incorporate biological relationships and environmental variation to strengthen interpretability. By combining multi-omics information, mixed-model results, and parental phenotypes, hybrid prediction approaches improve the transferability of models across different populations and breeding programs.

Overall, multi-omics integration spans genomics, transcriptomics, metabolomics, enviromics, and even trait-based transfer learning. It represents one of the most promising frontiers in modern breeding (Figure 3; Table 3). However, it also introduces challenges of dimensionality, data quality, and interpretability. As omics data generation becomes more routine, multi-omics prediction frameworks are expected to play a central role in future breeding, particularly for complex traits related to quality, nutrition, and stress tolerance that involve multi-layered regulatory control.

**TABLE 3:** Multi-omics integration strategies and representative methods in plant GP.

Type	Omics combined	Example model/Study	Species and task	References
Feature-level fusion	Genomic + Transcriptomic	Combined SNP and transcript feature models	*Maize* (complex traits like flowering time)	Azodi et al. (2020)
Intermediate joint fusion	Genomic + Phenomic (UAV imagery)	Multi-modal DL framework	*Wheat* (yield prediction)	Togninalli et al. (2023)
Ensemble/prediction-level fusion	Genomic + any other omics	Ensemble of individual predictors	General (strategy applied in various multi-omics studies)	Picard et al. (2021)
Hierarchical integration (knowledge-driven fusion)	Genomic + Transcriptomic + Metabolomic	COGCN learning on genomic, transcript, metabolite network	*Maize* (multi-omics trait prediction)	Li et al. (2025)

## 4 Transfer learning, multi-task modeling and foundation in genomic prediction

### 4.1 Cross-population and cross-species transfer in genomic prediction

A rapidly expanding area of GP research emphasizes using models trained in one population or species to improve predictions in another. This approach applies transfer and multi-task learning to plant breeding, offering new ways to enhance prediction for crops or breeding programs with limited data or few years of phenotyping.

One study showed that GP can be improved across species by drawing on knowledge from well-studied model plants. For example, using information from *Arabidopsis thaliana* to support the prediction of specialized metabolism genes in *tomato*, researchers improved model accuracy from an F-measure of 0.74–0.92 with *A. thaliana*-based predictions. This highlights how knowledge transfer from information-rich to less-studied species can significantly improve gene function inference and expand the utility of GP frameworks across species (Moore et al., 2020). ML provides a useful way to support orphan crops by drawing on genomic information from well-studied major crops. Using shared gene functions across related species improves prediction accuracy and speeds up breeding efforts in scenarios with limited data and resources (MacNish et al., 2025).

A common strategy in these approaches involves pre-training a deep neural network on a large source dataset, and fine-tuning it on a smaller target dataset representing a specific breeding population. In this framework, the lower layers capture broad polygenic signals and linkage disequilibrium structure, whereas the upper layers are adjusted to account for population-specific allele frequencies, correlations among traits, and patterns of environmental adaptation.

In summary, these results demonstrate that transfer learning enables the application of predictive models across diverse populations and species, reducing the need for extensive retraining and accelerating genomic-assisted breeding in both major and under-researched crops.

### 4.2 Multi-task and meta-learning strategies for knowledge transfer

The newly suggested MtCro framework takes this idea even further by using multi-task DL on different crops. This enhances the performance of multi-trait GP while reducing the reliance on extensive phenotypic records (Chao et al., 2025). A transfer learning system was established to forecast sorghum biomass. This model included genomic, meteorological, and UAV-based phenomic data, successfully capturing cross-season variance by integrating genotypic and environmental time-series characteristics using an LSTM-based recurrent network. It also showed that transferring pre-trained models between years can preserve high prediction accuracy with minimal field data (Wang T. et al., 2023).

Building upon recent advancements, meta-population and meta-learning frameworks are being investigated as broader paradigms. When linked populations share large QTL or allele effects, meta-population learning trains them all at once and increases the prediction accuracy. By teaching models how to quickly adjust to new crops or surroundings with only a small amount of labeled data, meta-learning takes this principle even further. This is like “learning how to learn.” In plant breeding, these algorithms could use historical datasets to provide genetic predictions for new species or populations.

These multi-task, transfer, and meta-learning methods showed a shift from predicting one trait to using integrated, knowledge-based genomic modeling. The ultimate goal is to create a single framework for all crops, conditions, and traits.

### 4.3 Cross-trait and foundation model approaches

GP is transitioning from task-specific models toward broader frameworks with shared patterns across traits and species. Conventional frameworks, which train separate models for each dataset, often fail to exploit linked biological information. Instead of training separate models, new approaches use shared learning to identify genetic patterns applicable across different traits.

Agricultural Natural Transformer (AgroNT) introduced a large-scale Transformer model trained on 48 edible plant genomes. Genomic, regulatory, and expression features were integrated to predict promoter and terminator activity and tissue-specific expression levels. The team also released the Plant Genomic Benchmark (PGB) as a standard dataset for developing and testing DL models, positioning AgroNT as a versatile foundation for genomic studies across plant species (Mendoza-Revilla et al., 2024). Because crop traits are interdependent, improving one trait may alter others. Recent studies have classified existing Multi-Trait Genomic Prediction (MT-GP) models and provided comparative evaluations using diverse rice traits under breeding-relevant scenarios (Mbebi et al., 2025). These results collectively reflect the expanding potential of AI-driven genomic models to unify multi-trait, multi-omic, and interspecies datasets within a single analytical framework.

Foundation-style genomic models might change predictive breeding in the future by acting as pre-trained backbones that can be used with different crops and settings. These frameworks hold promising potential to cut down on the amount of data needed for crops that do not have enough resources and speed up the process of finding genotype-to-phenotype links by putting cross-traits and cross-species relationships directly into model design. However, using foundation-style models in plant breeding is also exposed to several drawbacks, such as high computing costs and the possibility of bias from changes in data distribution and adaptability to specific environments (Li et al., 2024b; Chang et al., 2024).

**FIGURE 4:**
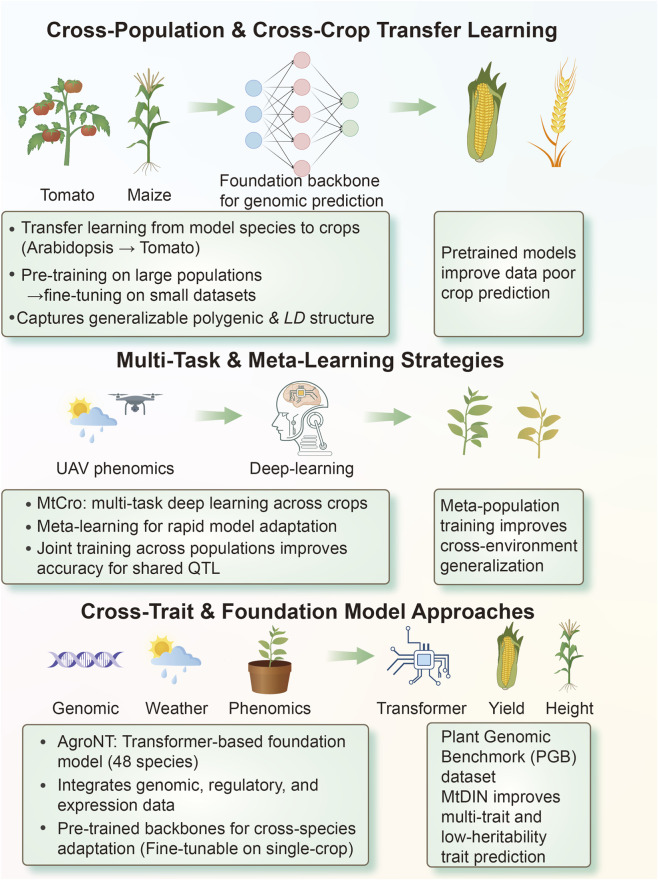
Cross-Population, Multi-Task/Trait, and Foundation Model Strategies in Genomic Prediction. Cross-population and cross-crop transfer learning improves prediction in data-poor crops using pre-trained deep networks from well-characterized species and large populations. Multi-task and meta-learning strategies combine UAV phenomics and deep-learning to train jointly across populations and environments, increasing accuracy for shared QTL and cross-environment generalization. Cross-trait and foundation model approaches, exemplified by AgroNT and MtDIN trained on benchmark datasets, integrate genomic, environmental and phenomic information to enable cross-species, multi-trait and low-heritability trait prediction.

As multi-omic and phenomic datasets continue to rise, these massive, generalizable models are poised to become the central infrastructure for next-generation predictive agriculture, bridging the gap between data richness in major crops and data scarcity in emerging species (Figure 4).

Several adaptation strategies explored in other foundation-model domains may be relevant for addressing these limitations. These include domain-adaptive pretraining or fine-tuning on target species or breeding populations, parameter-efficient fine-tuning approaches that update only a subset of model parameters, and few-shot calibration using limited labeled data. In addition, compute-aware strategies such as model distillation, parameter sharing, and modular architectures have been proposed to reduce training and deployment costs in large language and vision models. While these approaches have not yet been systematically evaluated in large-scale breeding programs, they outline plausible and transferable directions for adapting foundation-style genomic models to data-limited crops and environment-specific prediction tasks.

### 4.4 Biological validation and consistency with known genetic evidence

Although AI based GP models are primarily optimized for predictive accuracy rather than causal inference, an important question is whether the signals emphasized by these models are consistent with established genetic and biological knowledge. To assess this, recent studies have compared model-derived feature importance, with previously reported quantitative trait loci (QTL), expression quantitative trait loci (eQTL), and curated biological pathways.

Grain size is a key yield component in cereal crops and exhibits conserved genetic control across species. A large-scale GWAS in sorghum identified 81 grain size-associated QTL across multiple environments, many of which were enriched for orthologues of known grain size genes in rice and maize. By applying a panicle-halving treatment during flowering to reduce variation in grain filling, this study preferentially captured genetic effects related to intrinsic grain size potential. The GS3 gene family (Grain Size 3) shows strong concordance with previously reported grain size-associated QTLs. Multiple studies have demonstrated that GS3 and its orthologs in model and crop species, including ZmGS3 in maize and SbGS3 in sorghum, play conserved roles in regulating grain length and width (Tao et al., 2020).

In soybean, flowering time and maturity have been extensively dissected through classical QTL mapping using recombinant inbred line populations, revealing several major and pleiotropic QTL (e.g., on chromosomes 4 and 6) that control flowering, maturity, and reproductive period, and are embedded within well-characterized regulatory networks involving *E loci* and *FT* homologs (Kong et al., 2018).

High-resolution, open-access GWAS platforms provide a critical foundation for linking genetic variation to complex agronomic traits and for translating genetic discoveries into practical crop improvement under increasing global food demand (McCouch et al., 2016).

Together, these examples illustrate that a subset of signals highlighted by GP models overlap with well-established QTL and conserved regulatory genes across crops, providing empirical biological support for their predictive relevance.

## 5 Interpretability, reproducibility, and computational adoption in breeding

As modeling complexity and data integration increase, appropriate evaluation and validation become central to assessing the real-world utility of AI-based GP. In breeding applications, predictive performance must be interpreted in the context of forward prediction across years, environments, and populations, rather than random cross-validation alone. The following sections discuss key challenges related to evaluation, generalization, and deployment, which ultimately determine whether methodological advances translate into breeding impact.

It is widely acknowledged that AI and DL could be useful for GP, yet they will not be used in breeding programs unless they show that they work in practice. Plant breeders are practical; new strategies only become popular if they clearly make selection more efficient, increase genetic gain, or save time and money in real breeding cycles. Hence, in addition to typical measures like cross-validation accuracy, there is a growing focus on how well models work in real-world situations.

For instance, key requirements include prospective validation (e.g., forecasting future unphenotyped lines), year-to-year stability, and smooth integration into breeding selection processes for GS. In other words, an AI method has to show that it works in the real world, not simply in the past (Montesinos-López et al., 2021). To this end, a lot of breeders now test new prediction models by training them on data from the past (up to year N) and then checking the predictions against the results of the year N+1 trial. They aim to ensure that the model can really find the best prospects before phenotyping. An AI model repeatedly choosing genotypes that do well in the field gains credibility. Even small improvements in predicting ability can lead to faster genetic gain across generations, as each generation builds on the last. As a result, breeders are eager to try out AI methods, but only provided the tools are not too hard to use and the advantages are worth the cost of using them.

There are a number of practical things to be considered when moving AI-driven GP from research to everyday breeding. These factors, along with others, decide whether AI models can go beyond proof-of-concept and make real changes in how cultivars are developed and chosen.

**FIGURE 5:**
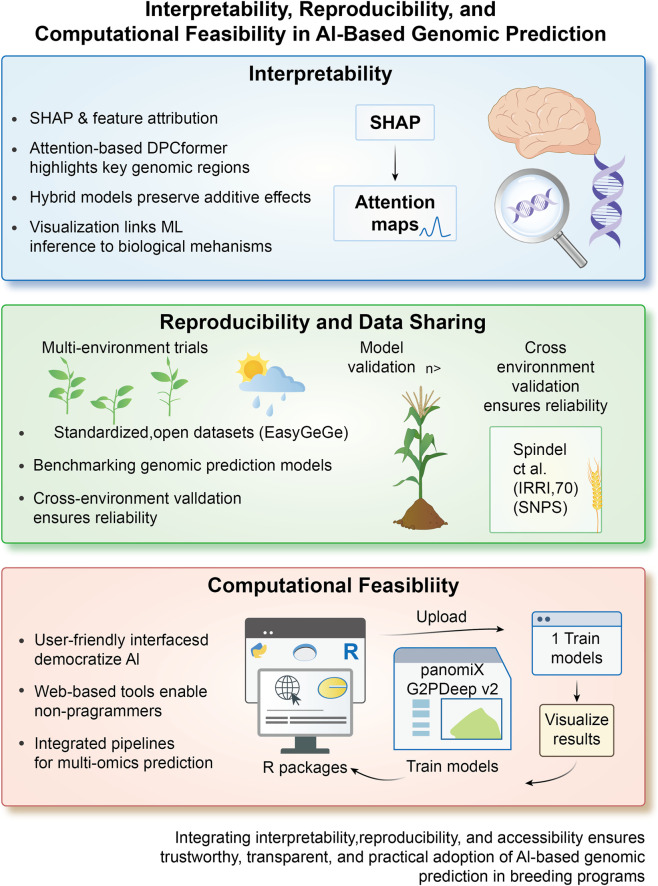
Interpretability, reproducibility data sharing, and computational feasibility in AI-enabled genomic prediction. Interpretability is enhanced via feature attribution methods, attention-based visualization, and approaches that link model behavior to underlying biological processes. Reproducibility is supported by multi-environment trials, publicly accessible standardized datasets, and cross-environment model validation. Computational feasibility is facilitated by user-oriented software interfaces, browser-based analytical tools, and integrated pipelines that streamline model training and multi-omics prediction.

The following subsections discuss recent progress and challenges related to interpretability, reproducibility, and computational considerations for adopting AI in plant breeding (Figure 5).

### 5.1 Interpretability

Model interpretability has become a central concern in applying AI to plant breeding. Unlike conventional linear mixed models (e.g., GBLUP) that provide clear outputs like marker effects or heritability estimates, modern DL frameworks often act as “black boxes”. Their internal logic is not readily transparent. To gain breeders’ trust and drive model adoption, prediction outputs should be biologically explainable and consistent with established domain knowledge (Wang J. et al., 2025). In this review, interpretability primarily refers to computational transparency (e.g., feature attribution and explanation of model behavior) rather than direct evidence of causal regulatory mechanisms. Therefore, model-derived explanations should be viewed as hypothesis-generating signals that require independent biological validation. Current GP models can be more interpretable when *post hoc* feature attribution methods such as SHAP are used. When three tree-based ML regressors were compared with traditional GP models (gBLUP and rrBLUP), their performance was assessed using Pearson’s correlation, R^2^, and Root Mean Squared Error (RMSE). SHAP values were applied to identify the most impactful SNPs on predicted phenotypes (boosting model transparency), and 10-fold cross-validation was used to validate model robustness (Novielli et al., 2024).

Researchers are coming up with ways to show how complicated models make predictions. Deep Pheno Correlation Former (DPCformer) is a GP model that captures complex genotype-phenotype correlations using CNNs and self-attention. It has been made possible by recent breakthroughs in DL. DPCformer performs better in predicting 13 features in five crops, such as maize, cotton, and tomato, compared to traditional approaches. Also, its attention method points out important genomic areas, giving biologically meaningful information about trait prediction and rendering it more useful for breeding programs (Crossa et al., 2025; Deng et al., 2025). Here, attention scores indicate which genomic segments are most informative for prediction under the learned representation, but they do not necessarily correspond one-to-one to known cis-regulatory elements (e.g., promoters/enhancers) without external annotation overlap and experimental validation.

Interpretability and reproducibility are becoming more and more important when creating AI-based GP models for plant breeding. Interpretability allows researchers to comprehend how the model associate genetic data with phenotypic results and to validate that its internal mechanisms have biological significance (Watson, 2022). Attention-based neural networks are a big step forward since they help the model find genomic areas that have the biggest effect on prediction accuracy. Hybrid models that integrate linear and nonlinear components retain the clarity of additive genetic effects (González-Camacho et al., 2018). Visualization helps make GP models more interpretable by converting outputs into biologically meaningful patterns and linking predicted traits to underlying genetic pathways. Approaches such as feature-weight maps, interaction networks, and co-expression modules further connect computational results with biological insight, improving the transparency and reliability of predictions (Conard et al., 2023). DL methods have demonstrated significant potential in elucidating intricate genetic patterns, while recent advancements in open-source software and data management have facilitated the wider implementation of GP and improved the efficacy of breeding operations (Crossa et al., 2025). A strong model should work the same way in different breeding populations and circumstances by using historical data to check predictions. Even little but steady early improvements over several breeding cycles can add up to big genetic benefits.

Researchers can strengthen confidence in AI-assisted decision making and achieve lasting improvements in crop performance by ensuring that model predictions are biologically interpretable and consistently.

### 5.2 Reproducibility and data sharing

In AI-assisted plant breeding, reproducibility involves not only read results but also maintaining consistent performance across diverse datasets, environments, and breeding cycles. The growing complexity and parameter richness of modern AI architectures underscore the increasing importance of confirming that their predictions are not artifacts of stochastic training processes.

One major difficulty is that there are few reliable public datasets for testing GP models. In plant breeding, data are often kept within institutions or companies. Thus, it may be challenging to compare or verify results from different studies. The lack of shared datasets makes it harder to check things on your own and makes it more difficult to see how well a model works with new germplasm. To solve these problems, new data-sharing projects and benchmarking platforms have appeared. The creation of shared repositories, including cooperative multi-environment trial datasets, enables direct comparison of GP models.

Previously, Spindel et al. conducted one of the initial field validations of GS in rice. They used 363 elite International Rice Research Institute (IRRI) breeding lines that had been genotyped with more than 70,000 SNPs. Their findings found that GP models were more accurate than pedigree approaches, involving values up to 0.63 for flowering time. The study confirmed the effects of marker density, genetic architecture, and model choice on prediction performance. This proves that GS can dependably make rice breeding more efficient (Spindel et al., 2015). Modern plant breeding has transitioned from conventional phenotype-based selection to predictive breeding that amalgamates genetic, phenomic, and environmental data. Combining these multidimensional datasets with AI-driven modeling makes it possible to speeds up genetic gain, and encourages smart, data-driven agriculture (Xu et al., 2022). Frameworks like EasyGeSe have been created to encourage consistent evaluation of genomic datasets, which is a big step toward open, reproducible AI in crop (Quesada-Trav et al., 2025).

Reproducibility also means that models should give stable and trustworthy results in different settings. If allele frequencies or trait topologies are different, a model works well on one breeding population may not work as well on another. To tackle this issue, researchers often use prospective validation, where models built on past data are tested on future breeding cycles to check their reliability and adaptability. Combining genomics, phenomics, and ML makes it easier to forecast complex traits and speeds up crop improvement, but there are still problems with data quality and model implementation (Yoosefzadeh-Najafabadi et al., 2025). Long-term breeding experiments show that advanced ML models such DL architectures may consistently improve prediction accuracy across cycles, even if the increases are small.

In short, to make AI-driven breeding more reproducible, endeavors are required to share data better, make tools available to everyone, and test models in real-world breeding situations.

### 5.3 Computational considerations for adoption

Another practical factor that affects adoption is how easy it is for users and how fast they work with computers. It is important for plant breeders to have easy-to-use software tools because many of them do not know much about Python/R programming or ML. In the early stages of GS, the accessibility of user-friendly programs (e.g., R packages) is a key driver of the rapid popularization of its models (Alemu et al., 2024). The rrBLUP R package improves GP accuracy by applying ridge regression and kernel-based approaches that account for both additive and nonadditive genetic effects. It has emerged as one of the most extensively used tools in plant breeding for estimating genomic breeding values and enhancing prediction accuracy for complex polygenic traits (Endelman, 2011). In the same way, current work has been focusing on incorporating ML approaches into interfaces that are easy for breeders to use. Accordingly, an open-source R package called “Sparse Kernel Methods (SKM)” has been developed. It uses a single framework to implement a number of well-known ML methods, including as gradient boosting, support vector machines, RFs, deep neural networks, etc., for GP. The SKM library focuses on making things easy (users can train and tune numerous ML models using the same syntax), which makes it easier for unexperienced individuals to try out complex models (Montesinos López et al., 2022).

Integrated platforms that bring together traditional and AI-based GP technologies are also starting to show up. A graphical interface or streamlined workflow is commonly used in these platforms to organize data, train models, and compare models. The objective is to facilitate breeders’ direct comparison of an additive genetic model against a DL model for a trait they prioritize, without requiring any coding effort. Early versions of these integrated tools have been said to simplify the adoption, basically “democratizing” AI in breeding by putting complex approaches into ready-to-use packages (Crossa et al., 2025). In addition, web-based applications are playing a role in accessibility. A notable example is G2PDeep-v2, a user-friendly web server introduced in 2024, which enables multi-omics phenotype prediction via DL (Zeng et al., 2024). Users can upload genomic and transcriptomic data through a web interface, select from various algorithms (from support vector machines to multi-layer CNNs), and let the server train and tune models on high-performance computing resources. The results (predicted phenotypes, important markers, etc.) are then visualized on the platform, and even follow-up analyses like gene set enrichment can be performed to help interpret the predictions. This kind of end-to-end platform lowers the barrier for non-programmers to leverage AI, as everything runs in the cloud with an intuitive interface. Another emerging toolkit is panomiX (developed as of 2025), which provides a pipeline for integrating different omics data and building predictive models for plant traits. PanomiX is designed as a “one-stop” toolbox where breeders can combine genomic, transcriptomic, metabolomic, and phenotypic data, and apply built-in ML pipelines (including feature selection and model training) through a user-friendly application (Sahu et al., 2025). By automating multi-omics data integration and model building, it aims to enable non-experts to uncover trait-gene relationships and improve predictions without needing to write complex code. All of these tool-building efforts, from R packages to web platforms, are helping to democratize AI methods in breeding by making advanced models accessible to practitioners. Table 4 summarizes the major DL architectures reviewed in this article, providing an integrative overview of current modeling strategies.

**TABLE 4:** Representative statistical and DL approaches for GP in plants.

Model/Method	Task	Data and species	Framework	Results/Estimation	Interpretability/Validation	Compared with GBLUP
DNNGP (Wang et al., 2023a)	Integrating multi-omics and environmental variables for phenotypic prediction	Multi-omics data (genotypes, expression, and environmental variables) across multiple crops	DNNs	Prediction accuracy (r, 10-fold CV): DNNGP outperformed GBLUP at n = 100 (e.g., TKW 0.51 vs. −0.01) and remained superior at n = 2000 (e.g., TKW 0.67 vs. 0.18)	10-fold cross-validation with sensitivity analyses (sample size, DR); interpretability assessed using density/contour plots and ODR to capture non-linear relationships	DL captures complex nonlinear interactions and can outperform GBLUP, whereas GBLUP is more robust under predominantly linear effects
SoyDNGP (Gao et al., 2023)	Prediction of soybean traits using GP/GS (GP/GS)	Soybean genotype sequences	1D CNN	High accuracy across traits (e.g., FC 0.94, PDENS 0.85, POD 0.83, ST 0.82), comparable to DNNGP/rDeepGS and clearly better than mDeepGS (e.g., FC 0.67). For regression, SoyDNGP showed higher Pearson r and ∼10× lower MSE than DNNGP (DNNGP r ∼5% lower)	Validated by 10-fold cross-validation and tested across different datasets/populations to assess robustness and generalization; interpretability mainly assessed via predicted-observed distribution patterns	DL can surpass GBLUP with strong local structure, but is not necessarily superior when G × E effects dominate
PNNGS (Xie et al., 2024)	Capturing fine-scale and coarse-scale patterns of genomic markers	Wheat and related crops	Parallel CNN	Pearson r (primary; NRMSE secondary), 10-fold CV. Across 24 trait-dataset cases (rice/sunflower/wheat/maize), PNNGS outperformed RRBLUP/RF/SVR and improved over DNNGP by +0.031 r on average	10-fold CV with fold-wise stability checks (e.g., GL r = 0.688–0.795, SD 0.032). Robustness addressed via PCA + K-means clustering and stratified sampling for unbalanced/non-i.i.d. data; interpretation mainly via phenotype distribution pattern analyses	DL excels in capturing multi-scale associations and outperforms GBLUP under strong nonlinear signals, whereas GBLUP is more robust in small-sample settings
WheatGP (Wang et al., 2025a)	Simultaneous modeling of short- and long-range genetic dependencies in wheat	Multiple traits in wheat	CNN + LSTM Hybrid	Outperformed rrBLUP/XGBoost/SVR/DNNGP, achieving yield accuracy ∼0.73 and 0.62–0.78 across agronomic traits	Validation: 10-fold CV (limited wheat sample sizes); additional robustness checks include MAE/MSE comparisons (e.g., transfer-learning setting) and tests on other crops/multi-omics datasets. Interpretability: SHAP used to quantify input/marker contributions	DL outperforms GBLUP in long-range dependency tasks, whereas GBLUP may be more robust when G × E effects are explicitly modeled
GCN and GCN-RS (Kihlman et al., 2024)	Construction of individual relationship graphs for GP (non-Euclidean structures)	Population relationship graphs	GCNs	GCN-RS achieved the best distance correlation across all datasets (QTLMAS 0.411, Wheat 0.496, Mice 0.404, Pig 0.447) and the top Pearson r in 3/4 datasets (QTLMAS 0.431, Mice 0.384, Pig 0.497; Wheat 0.456, second to BRKHS 0.477)	Hold-out validation: 100 individuals as test set; remaining data split into 80% train/20% validation. Robustness assessed by varying train/test proportions (90/10→50/50) (Table 4). Interpretability: graph edges defined by kNN (Euclidean distance in marker space)	DL excels in complex non-Euclidean networks, while GBLUP is more robust with small samples
HGATGS (He et al., 2025)	Crop GS through modeling higher-order similarity patterns	Hypergraphs constructed from inter-sample similarity	HGATs	Validation: 5-fold CV. Best (HGATGS/HGCN + ResNet + Attention): r = 0.53 (Wheat 599), 0.67 (Wheat 2000), 0.90 (G2F 2017), outperforming baseline variants in Table 4	Validation: 5-fold CV with shuffled splits; ablation of attention and residual modules to verify component contributions. Interpretability: attention/hypergraph structure analyzed mainly via visual/statistical pattern analyses (e.g., cosine-distance distributions); no single interpretability score reported	DL excels with nonlinear similarity structures; GBLUP is more robust with small samples or weak signals
SNP2Vec (Cahyawijaya et al., 2022)	Learning SNP contextual representations and uncovering gene–gene associations	Single-nucleotide sequences	Transformer-based embedding models	10-fold CV; metrics = Accuracy, AUROC, AUPRC. Dipformer (SNP2Vec) achieved Acc 0.643/AUROC 0.673/AUPRC 0.734, outperforming DeepSEA (0.591/0.579/0.703), PLINK PRS (0.592/0.607/0.705), and Hapformer (0.572/0.615/0.715)	Validation: 10-fold cross-validation on a Chinese LOAD cohort; evaluated under different input-length settings (APOE-only, APOE+10k, APOE+20k). Interpretability: no dedicated interpretability results reported; interpretability (attention analysis/gradient saliency) is discussed mainly as future work	Transformers excel under strong long-range dependencies, whereas GBLUP is more robust with limited data or explicit G × E modeling
GPTransformer (Jubair et al., 2021)	Modeling SNP sequences to improve predictive performance	SNP sequences with large sample sizes	Transformer	Across 3 train/val/test splits, GPTransformer achieved r = 0.748 (DON) and r = 0.703 (FHB); BLUP achieved r = 0.789 (DON) and r = 0.681 (FHB) (differences not significant: p = 0.576 for DON; p = 0.639 for FHB)	Validation: evaluated with three independent train/validation/test splits; test-set stability reported (SD r = 0.04 (FHB), 0.09 (DON) vs. BLUP 0.008/0.04). Interpretability: limited-authors note ML/DL models are opaque	DL excels in long-sequence tasks with strong nonlinearity and sufficient data; GBLUP is more robust with small samples
EBMGP (Ji et al., 2025)	Extraction and selection of short- and long-range connectivity features	High-dimensional SNP data	ElasticNet + BERT	EBMGP ranked best in 13/16 tasks, delivering +0.74% to +9.55% accuracy gains over the second-best model; e.g., for soybean traits MS/NMSP, EBMGP exceeded BayesB by +2.90%/+1.09%, while GBLUP was higher for VE by +6.52%	Validation: 5-fold cross-validation with robustness assessed via fold-wise SE of prediction accuracy. Interpretability: Elastic Net feature selection (sparse SNP subset) plus multi-head attention pooling provides feature weighting, but no explicit interpretability score is reported	DL combined with feature selection shows strong performance
DeepPlantCRE (Wu et al., 2025)	Prediction of genomic regulatory elements such as promoters and enhancers	Crop genomic sequences	Transformer + CNN	Accuracy/AUC-ROC/F1 (mean ± SD) across 5 crops: DeepPlantCRE achieved Acc 80.7%–90.9%, AUC 86.2%–96.6%, F1 76.1%–93.4% (Table 1; e.g., cotton Acc 90.9%, AUC 96.6%, F1 93.4%) and showed strong cross-species transfer with accuracy up to 92.30%	Validation: chromosome-wise k-fold CV (k = number of chromosomes per species) + cross-species cross-validation/transfer experiments. Interpretability: DeepLIFT importance scores + TF-MoDISco motif discovery; learned motifs matched known TFBS in databases (e.g., JASPAR), supporting biological interpretability (no single interpretability score)	Enhances interpretability, lacks direct task-level comparability, and shows promise as a DL backbone
G2PT (Lee et al., 2025)	Multimodal genomic and environmental time-series modeling for trait prediction	Genotype and environmental sequences across multiple time points	Bidirectional LSTM + Transformer	Explained variance (R^2^) under 5-fold nested CV (UK Biobank): TG/HDL ∼0.18–0.19, LDL ∼0.09–0.10, T2D ∼0.043–0.047; external transfer (UKB→All of Us) for T2D retained R^2^ = 0.025	Validation: 5-fold nested cross-validation + external cohort transfer (All of Us). Interpretability: attention-based gene/system importance (correlating attention with predicted phenotype) and epistasis search within top-ranked systems	GBLUP suits additive effects; Transformers excel with complex interactions
NetGP (Zhao et al., 2025)	Multi-omics GP through integration of gene networks and DL	SNPs, gene expression, and networks across multiple species	Network and Cross-omics GCN	NetGP achieved top performance across 11 rice traits in genomic/transcriptomic/multi-omics prediction; e.g., GP reached HD 82.63%, PH 84.67%, SL 80.92%, with gains over GenNet of +3.28% to +18.63% (largest for GT)	Validation: 10-fold cross-validation (random split into 10 folds; each fold used once as test). Robustness further checked via feature-selection comparisons (PCS vs. PCA/LD). Interpretability: gene-network–based architecture (genes as nodes; edges defined/expanded via network design) provides structural interpretability	GBLUP is more reliable in small SNP datasets, while DL models become superior upon the incorporation of network-level
COGCN (Li et al., 2025)	Capturing higher-order interactions among genomics, transcriptomics, and metabolomics	Maize multi-omics data	Cross-omics graph convolution	COGCN achieved 0.591 (Yield), 0.567 (Height), 0.651 (Flowering time), outperforming SVR/RF/GBLUP/BayesB/RRBLUP/DEM on the maize multi-omics dataset.	Validation: 5-fold cross-validation + ablation (Table 2: COGCN-cross 0.556/0.529/0.623, COGCN-gcn 0.571/0.543/0.634, full COGCN 0.591/0.567/0.651). Interpretability: model is structurally interpretable via cross-omics tensor + GCN integration	Multi-omics integration with high interaction complexity substantially improves multi-trait prediction, whereas GBLUP is more stable with additive SNPs alone
MtCro (Chao et al., 2025)	Multi-trait (and multi-environment) GS	Bread wheat (*Triticum aestivum*), yield across 4 environments (ENV1–ENV4) treated as 4 phenotypes	MoE: MLPs + gating network + genotype + PCA	Metric: Pearson correlation (predicted vs. observed; MSE also analyzed). MtCro > DNNGP/SoyDNGP with gains of ∼1–9% (Wheat2000), 1%–8% (Wheat599), 1%–3% (Maize8652); overall ∼2–3% consistent improvement for multi-phenotype prediction	Validation: cross-validation; robustness assessed via 5-fold CV and multiple parameter initializations, reporting SD (Pearson)/SD (MSE) (Tables 2, 3). Interpretability: phenotype-correlation heatmaps + predicted vs. observed distribution/linear-fit (e.g., BLUE/regression across environments); no single interpretability score	GBLUP is more stable in small-sample settings, whereas DL models outperform in multi-task scenarios
AgroNT (Mendoza-Revilla et al., 2024)	Sequence-based prediction of genomic regulatory features	Whole-genome DNA sequences from 48 plant species, multi-task training using diverse regulatory and transcriptomic datasets	Transfer learning and task-specific fine-tuning	Achieves state-of-the-art performance across multiple regulatory annotation and expression prediction tasks; enables large-scale *in silico* saturation mutagenesis (>10 million variants) and accurate prioritization of regulatory variants	Interpretability: Model interpretability assessed through *in silico* saturation mutagenesis and systematic analysis of sequence-level variant effects. Validation: Validation performed across multiple plant species and tasks cross-species	GBLUP targets breeding value estimation from genotype-phenotype data, whereas AgroNT provides transferable sequence representations for regulatory annotation rather than direct GP.
DPCformer (Crossa et al., 2025)	GP of complex quantitative traits	SNP genotype data from crop breeding populations, primarily maize	Dual-path Transformer architecture with parallel local and global attention modules	Across five crops and multiple traits, DPCformer reports PCC/accuracy of 89.12% (DTT), 68.93% (PH), 91.41% (EW) in maize (Henan) and 93.50% (DTT), 76.24% (PH), 93.01% (EW) in maize (Beijing); achieves 84.45% PCC for rice plant height (n = 530), 74.19%/71.45%/74.85%/72.30% for cotton fiber traits (FE/FL/FS/FM), 73.49% PCC in tomato (n = 332), and 62.12%/56.87%/65.96%/65.96% across chickpea traits; ablation shows PCC improving from 0.8376 → 0.9076	Interpretability: Interpretability is inferred from attention weight distributions, highlighting informative markers and long-range genomic dependencies in an implicit manner. Validation: Validation is performed using cross-validation across traits and populations, demonstrating stable performance across independent test sets	GBLUP is robust under additive assumptions and small sample sizes, whereas DPCformer captures nonlinear and long-range dependencies and performs better in complex, multi-trait settings

In real-world deployments, one pragmatic strategy has been to augment rather than replace existing selection methods with AI models. For instance, a breeder might use a state-of-the-art DL model to analyze genomic data and flag a subset of candidate lines that are predicted to have large non-additive gains (e.g., specific combining ability or heterosis potential). DL has emerged as a promising alternative to conventional GP methods by offering greater flexibility to model complex and nonlinear relationships between genotypes and phenotypes. Although its predictive advantage over traditional models is not yet consistently demonstrated, DL shows strong potential for integrating multi-source omics data and improving prediction accuracy when large, high-quality training datasets are available. In this way, the deep model is adding value by uncovering patterns that the linear model might miss, but it is not solely responsible for all decisions. This blended approach hedges against the risk of any single model being wrong and eases the transition to new technology, breeders see tangible benefits (like faster gain or better targeting of special trait combinations) without having to abandon their familiar tools. Over time, as confidence in the AI predictions grows, the community consensus is that independent validation and interpretability are key to earning trust in AI, and that gradual integration, using AI as an aid initially, and expanding its role as it proves reliable is a sound path forward.

To sum up, AI-driven GP is moving from being used in research to being used in real-world breeding. Studies have shown that when enough data and computing power are available, DL models often outperform traditional methods. At the same time, researchers are working to make these models easier to understand and more reliable by improving transparency, encouraging data sharing, and developing common benchmarking practice. The availability of user-friendly software that fits easily to use helps these methods reach wider use. As the tools become more refined, the gap between theory and practice is expected to narrow, laying the groundwork for data-driven breeding strategies that improve crop improvement more efficiency.

## 6 Discussion

Recent advances in AI-driven GP are transforming the field from marker-based association toward integrated modeling of genetic, molecular, and environmental processes. Deep neural architectures, graph-based networks, and attention mechanisms now enable the capture of nonlinear epistatic interactions and long-range genomic dependencies that were previously inaccessible to linear mixed models. These developments mark a conceptual shift from fitting additive genetic effects to learning hierarchical representations of plant biology.

A second frontier of progress lies in multi-omics fusion and enviromic modeling. Combining genomic data with transcriptomic, metabolomic, phenomic, and environmental layers has steadily improved prediction accuracy for complex traits. Yet most frameworks still merge information statistically, rather than modeling the biological cascades that regulate gene expression and shape developmental timing. Scale mismatches, variable noise levels, and uneven sampling resolution across omics tiers continue to inject uncertainty. Tackling these problems will demand coordinated experimental designs, cross-omics anchors such as eQTL networks, and phenotyping that captures both spatial and temporal dimensions.

Finally, moving from theory to routine breeding use is still the main hurdle. Prediction accuracy has improved, yet breeders will only embrace models that deliver consistent results across seasons and sites, explain their outputs in plain terms, and slot cleanly into daily decision-making. FAIR-compliant data, open benchmarking platforms, and intuitive software are steadily narrowing the distance between proof-of-concept and field application.

However, scaling large models, ensuring cross-population generalization, and avoiding bias remain unresolved.

1. From statistical association to mechanistic understanding for causation

Existing GP frameworks mostly rely on correlations and leave the regulatory or causal routes that connect variants to phenotypes implicit. Although attention layers, graph neural networks and multi-omics fusion lift predictive performance, they still overlook the gene-regulatory wiring, chromatin architecture and spatiotemporal boundaries that govern when and how traits appear. Weaving these biological structures into the model will be a prerequisite for shifting GP from pattern matching to predictions that reflect mechanism.

2. Toward biologically grounded unified multi-omics and phenomics modeling

Combining genomic data with transcriptomic, metabolomic, and environmental information has sharpened predictions for complex traits, yet the dominant models still fuse statistics rather than trace how molecular states unfold during development or react to external cues. Disparities in data magnitude, sampling noise, and temporal resolution continue to block progress. To capture the full genotype-environment-phenotype continuum.

3. Practical deployment, robustness, reproducibility, and trust

To embed AI-driven GP in routine breeding, models must deliver stable accuracy across seasons, locations and germplasm. Reproducibility is still constrained by sparse public benchmarks, divergent preprocessing pipelines, and software that rarely turns predictions into clear selection instructions for breeders. Independent validation and FAIR-compliant data resources will be essential for credible benchmarking under field conditions. Foundation-style models could extend across species, yet their practical use hinges on solving computational expense, data bias and uneven environmental adaptability first.

**FIGURE 6:**
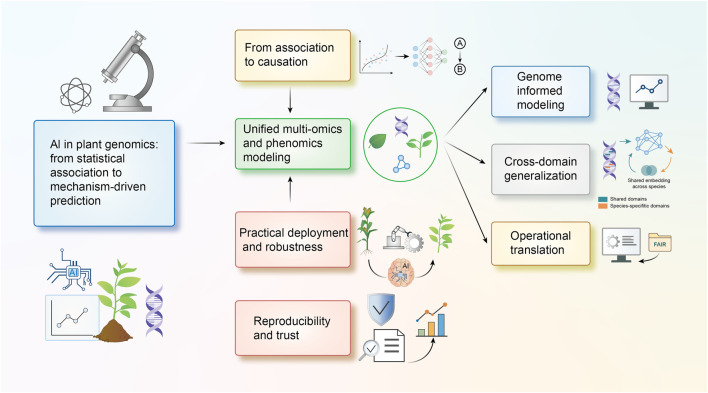
Framework shaping next-generation plant genomic prediction. Recent developments in plant genomic prediction are converging toward a unified framework that links biological understanding with practical deployment. The framework highlights the shift from statistical association toward causation-oriented modeling, the integration of multi-omics and phenomics information within coherent predictive systems, practical and robustness for practical breeding use, and reproducibility with trust form an additional foundation for reliable model deployment.

Taken together, bridging the gap between accuracy, mechanistic insight, and operational reliability will determine whether AI-driven prediction becomes a routine component of breeding programs. The next-generation of GP frameworks will likely combine mechanistic modeling, multi-omic integration, and large-scale transfer learning to build scalable, interpretable, and climate-resilient predictive systems (Figure 6). Key challenges and methodologies are further elaborated in Boxes 1–5.

Box 1 **Broader risks and boundary conditions of AI-based genomic prediction.**

- Domain shifts: Domain shift severely limits the transferability of DL models for crop yield prediction, as models trained in one region often perform poorly when applied to other regions with different yield distributions. Although unsupervised domain adaptation (UDA) can partially alleviate this issue by aligning feature distributions, its common assumption of identical label spaces is frequently violated in heterogeneous agricultural systems, leading to negative transfer. By explicitly accounting for label space mismatch, a partial domain adaptation framework achieved mean R^2^ values of 0.70 for corn and 0.67 for soybean (2019–2021), outperforming conventional ML and UDA models by 6%–46% (Ma et al., 2023). In agricultural image analysis, domain shift has emerged as a central challenge limiting model generalization across real-world scenarios. Differences in data acquisition environments, including sensor types, illumination conditions, seasonal variation, crop varieties, and soil backgrounds lead to substantial distribution discrepancies between source and target domains, even for the same task. As a result, models trained under controlled settings or in specific regions often experience severe performance degradation when deployed across regions, seasons, or field conditions, highlighting domain shift as a key barrier to the practical deployment of agricultural intelligent vision systems (Hu et al., 2025).
- Crop bias: Current AI research in agriculture is predominantly focused on a small number of staple crops, such as maize, rice, and wheat, while orphan and minor crops remain underrepresented due to limited data availability. This crop bias restricts model generalizability and overlooks diverse agricultural systems, particularly in developing regions where orphan crops are important for nutrition and stress tolerance. Recent work suggests that transferring knowledge from major crops to related orphan crops using ML can partially address these limitations. Expanding datasets and benchmarks to include underrepresented crops is therefore essential for more inclusive and generalizable AI-driven crop improvement (MacNish et al., 2025).
- Energy/carbon costs: The rapid growth in computational requirements for DL has led to substantial energy consumption and carbon emissions, raising concerns about the environmental sustainability of large-scale models. In addition to their environmental cost, the financial burden associated with training and deploying such models creates barriers for researchers with limited resources, particularly in emerging economies. These challenges highlight the need to consider computational efficiency and energy cost, alongside predictive accuracy, when developing and evaluating AI models (Schwartz et al., 2020). The explosive growth in ML computation has led to substantial energy consumption and carbon emissions, which can vary by up to 100–1,000× depending on model design, hardware, data center, and deployment choices, underscoring the need to evaluate AI models based not only on accuracy but also on energy efficiency and environmental impact (Patterson et al., 2021).
- Black-box generalization: Complex AI models in plant and agricultural research often lack clear interpretability, increasing the risk that models exploit dataset-specific correlations rather than biologically meaningful signals, which in turn undermines generalization under novel conditions (Mostafa et al., 2023). Without mechanistic grounding, data-driven crop models risk black-box generalization, leading to limited transferability and unreliable decision support under complex and changing agricultural conditions (Zheng et al., 2026).

Box 2 **AI-enabled modeling of stress responses under dynamic environments.**

- Stress‐responsive traits: Abiotic stresses such as drought, heat, and salinity induce distinct and measurable plant traits, including leaf rolling, stomatal conductance, canopy temperature, and root architectural responses. AI models should therefore focus on these stress-sensitive proxy traits rather than relying solely on final yield outcomes, and where possible incorporate biochemical indicators such as stress-related hormone levels. Collecting trait data under controlled stress conditions, for example, through greenhouse screening experiments, can enable AI-based mapping of intermediate phenotypes to stress tolerance. The identification and integration of such intermediate traits, including indicators like chlorophyll fluorescence for heat tolerance, can further improve model interpretability and robustness (He et al., 2023; Rico-Chávez et al., 2022).
- Time-series omics: Plant responses to abiotic stresses are dynamic processes that unfold over time, and static omics measurements fail to capture these temporal trajectories. Time-course transcriptomic and multi-omics data reveal how gene expression and regulatory networks change during stress exposure, as shown in longitudinal RNA-seq analyses under drought or salt stress that identify time-dependent patterns of gene activation and co-expression modules (Zhao et al., 2023; Yi et al., 2022). To model such dynamics, sequential approaches that account for temporal dependency are needed, enabling more accurate inference of stress response mechanisms and regulatory interactions from time-series omics data.
- Latent stress (early warning): Early detection of latent stress before visible phenotypic damage has been enabled by advances in imaging and sensing technologies. The integration of hyperspectral sensing with ML has enabled high-throughput identification and prediction of early stress markers, as shown in soybean under progressive water stress, where stress-related signals were detected before overt phenotypic decline (Oliveira et al., 2025). These findings are further supported by systematic reviews highlighting the broad potential of hyperspectral remote sensing for early detection of plant stress and disease across diverse agricultural scenarios (Terentev et al., 2022).

Box 3 **Realistic Implementation of Multi-Omics in Breeding Programs.**

- Challenge: Multi-omics data (including transcriptomics and metabolomics) can significantly enhance predictive accuracy in breeding programs, but these data are not always available in routine breeding pipelines. Collecting multi-omics data from large breeding populations can be resource-intensive, and often, only genomic data are routinely available.
- Solution: To realistically implement multi-omics integration, breeders could start by combining genomics with easily accessible phenotypic and environmental data, leveraging simpler models that focus on specific, measurable traits. Transfer learning from species with extensive multi-omics datasets (such as Arabidopsis or rice) can be applied to crops with limited omics resources. Additionally, graph-based models and multi-omics fusion techniques can be employed to integrate the data that is available, even if it is partial or noisy. Breeders can also prioritize the integration of genomic and phenotypic data as a first step before scaling up to full multi-omics integration.
- Future Consideration: As high-throughput multi-omics platforms become more common, more crop-specific, multi-omics datasets will be available, which can enhance prediction accuracy and decision-making. However, ensuring data standardization and improving computational pipelines will be key to making these approaches feasible at scale.

Box 4 **Balancing Model Complexity with Operational Simplicity.**

- Challenge: Advanced AI models are powerful but often complex and computationally demanding, which can be a barrier for routine use in breeding pipelines.
- Solution: The future of AI in breeding lies in simplifying operations while maintaining complex predictions. AI tools should be intuitive and easy to use, enabling breeders at all skill levels to make data-driven decisions without needing advanced technical knowledge. User-friendly platforms will allow breeders to easily apply complex models, ensuring accessibility and reducing the gap between users with different expertise levels.
- Human-AI Collaboration: AI should serve as a support tool, not replace human expertise. By simplifying model usage, breeders can focus on decision-making, while AI handles the data complexity, making advanced tools accessible for all.

Box 5 **Conceptual Integration of Methodologies.**

- Comparison of Models: As shown in Tables 1, 4, different AI models have distinct advantages and limitations. Linear models, like GBLUP, are simple and interpretable but struggle with complex, non-linear gene-environment interactions. On the other hand, DL models capture these complexities well but require large datasets and are less interpretable.
- Emerging Techniques (GNNs and Multi-Omics): Graph Neural Networks (GNNs) and multi-omics integration show promise for capturing gene regulatory networks and improving prediction accuracy. However, their practical application is still emerging and requires further refinement.
- Gaps and Future Directions: A key gap in current research is the lack of integrated approaches that combine the strengths of these models. For instance, using linear models for early-generation selection and DL or GNNs for more complex traits could balance prediction accuracy and operational simplicity.

## 7 Conclusion and future perspective

With the development of these components, AI-driven GP will transition from a theoretical framework to the foundational basis for predictive breeding decisions. These systems will speed up genetic gain, and help farming stay viable in a changing climate.

Ultimately, the future of plant breeding depends not only on the ability to predict traits but also on understanding and engineering the genetic mechanisms that sustain productivity and adaptive capacity.

## Glossary

### Genetic effects and quantitative genetics

AA: Additive × Additive effect
AD: Additive × Dominance effect
DD: Dominance × Dominance effect
G × E: Genotype × Environment interaction

### Genetic loci and omics concepts

QTL: Quantitative Trait Locus/Loci
eQTL: Expression Quantitative Trait Locus
SNP: Single Nucleotide Polymorphism

### Classical genomic selection and prediction models

GP: Genomic Prediction
GS: Genomic Selection
GBLUP: Genomic Best Linear Unbiased Prediction
RR-BLUP/rrBLUP: Ridge-Regression Best Linear Unbiased Prediction
RKHS: Reproducing Kernel Hilbert Space
BGLR: Bayesian Generalized Linear Regression
LASSO: Least Absolute Shrinkage and Selection Operator
SKM: Sparse Kernel Methods
PCS: Pearson-Collinearity Selection

### Multi-omics and metabolomics-based prediction

MM-GP: Metabolic Marker-Assisted Genomic Prediction
MT-GP: Multi-Trait Genomic Prediction
MGBLUP: Metabolomic-Genomic Best Linear Unbiased Prediction
NMR: Nuclear Magnetic Resonance (metabolomics spectroscopy)

### General ML and DL methods

AI: Artificial Intelligence
ML: Machine Learning
DL: Deep Learning
RF: Random Forest
SVR: Support Vector Regression
CNN: Convolutional Neural Network
RNN: Recurrent Neural Network
LSTM: Long Short-Term Memory
Bi-LSTM: Bidirectional Long Short-Term Memory

### DL-based genomic prediction and selection

DNNGP: Deep Neural Network Genomic Prediction
PNNGS: Parallel Neural Network for Genomic Selection
HGATGS: Hypergraph Attention Network for Crop Genomic Selection
EBMGP: Elastic BERT Model for Genomic Prediction
NetGP: Gene Network Prediction model

### Transformer-based and representation learning models

AgroNT: Agricultural Natural Transformer (large-scale Transformer model pretrained on multi-crop datasets)
G2PT: Genotype-to-Phenotype Transformer
GPTransformer: Genotype-Phenotype Transformer
TrG2P: Transfer-learning Genotype-to-Phenotype framework
DPCformer: Deep Pheno Correlation Former (interpretable DL model)

### Graph-based neural networks

GCN: Graph Convolutional Network
GNN: Graph Neural Network
COGCN: Cross-Omics Graph Convolutional Network

### GWAS-related analytical frameworks

GWAS: Genome-Wide Association Study/Studies
DL-GWAS: Deep Learning-based Genome-Wide Association Study
ML-GWAS: Machine-Learning Genome-Wide Association Study

### Interpretability and evaluation metrics

SHAP: SHapley Additive exPlanations
RMSE: Root Mean Squared Error

### Data resources, benchmarks and principles

AgERA5: Agricultural ECMWF Reanalysis v5 (environmental dataset)
PGB: Plant Genomic Benchmark
FAIR: Findable, Accessible, Interoperable, Reusable (data principles)

### Agricultural research organizations

CIMMYT: Centro Internacional de Mejoramiento de Maíz y Trigo (International Maize and Wheat Improvement Center)
IRRI: International Rice Research Institute

### Phenotyping platforms and tools

UAV: Unmanned Aerial Vehicle

### Biomedically derived indicators

TG/HDL: Triglyceride-to-High-Density-Lipoprotein
