# Supplementary material for: Beyond QTL and GWAS: how deep learning, graph models, and multi-omics are reshaping plant genomic prediction analysis
Source: Front Genet. 2026 Apr 17;17:1783939. doi: 10.3389/fgene.2026.1783939 (PMC13132505; doi:10.3389/fgene.2026.1783939)
Supplement: Supplementary file 1 [file Supplementaryfile1.docx]

**Appendix A: Proposed Modeling Strategies for Integrating Causal Biological Structures into AI**

1. **Gene Regulatory Networks + Graph Neural Networks (GNNs)**
   Encode gene regulatory networks (GRNs) as graphs, where nodes represent genes (or markers) and edges represent regulatory links. A GNN propagates information across these edges, capturing causal relationships between genes and their downstream effects.
2. **Transformer/Attention Models**
   Use multi-head attention to capture long-range interactions and regulatory motifs within genetic data. This approach highlights key SNP–SNP interactions and provides interpretability by identifying causally important genomic regions.
3. **Multi-Omics Graph Models**
   Integrate heterogeneous omics data (genomic, transcriptomic, proteomic, etc.) into a unified graph. A Graph Convolutional Network (GCN) is used to learn how combined genetic and omics variation propagates to phenotypes, improving prediction accuracy.
4. **Causal Inference Models (Structural Equation Modeling / Bayesian Models)**
   Build explicit causal graphs among traits or genes using structural equation models (SEM) or Bayesian networks. These models allow for the representation of direct and indirect genetic effects and support “what-if” predictions under different conditions.
5. **Mechanistic/Physics-Informed Machine Learning (ML)**
   Embed crop physiology or growth models into machine learning algorithms. This hybrid approach helps explain genotype-environment interactions (G×E) and improves predictions for non-additive genetic effects.

**Appendix B: Experimental Validation Designs**

1. **Genome-Wide Perturbations (CRISPR/Cas9)**
   Use CRISPR/Cas9 to perturb genes and observe phenotypic changes. This allows validation of causal effects predicted by AI models by directly manipulating genes and studying downstream effects.
2. **eQTL and Mendelian Randomization**
   Integrate eQTL data to examine causal relationships between gene expression and traits. Mendelian randomization can be used to test whether changes in gene expression causally affect phenotypic traits.
3. **Genotype × Environment (G×E) Trials**
   Conduct multi-environment field trials to validate how genotype effects vary across different environmental conditions. This helps to model true causal effects under realistic breeding conditions.
4. **Cross-Species Transfer Learning**
   Leverage data from well-studied species (e.g., Arabidopsis, rice) to pre-train models and transfer learned knowledge to target crops with limited data, validating causal relationships in less-studied species.
