# Supplementary material for: Beyond QTL and GWAS: how deep learning, graph models, and multi-omics are reshaping plant genomic prediction analysis
Source: Front Genet. 2026 Apr 17;17:1783939. doi: 10.3389/fgene.2026.1783939 (PMC13132505; doi:10.3389/fgene.2026.1783939)
Supplement: Supplementary file 2 [file Supplementaryfile2.docx]

Reference grouping:

Group A — Classical statistical genetics & linear/mixed/kernels (BLUP/Bayesian/RKHS, etc.)

1, 2, 3, 4, 5, 6, 7, 8, 9, 65, 82, 83, 84

Group B — Conventional ML (non-DL) and general ML methodology

11, 85

Group C — Deep learning for genomic prediction/selection (CNN/RNN/GNN/hypergraph; multi-trait DL)

10, 13, 14, 15, 16, 17, 18, 19, 21, 22, 43, 55

Group D — Transformers, self-supervised genomic representation learning, and foundation-style models

20, 23, 24, 45, 47, 48

Group E — Multi-omics integration & graph/network-based multi-modal fusion for phenotype prediction

25, 27, 28, 29, 30, 31, 32, 33, 34, 35, 36, 39, 40, 67, 68

Group F — G×E / multi-environment modeling, transfer learning, domain adaptation, and remote sensing fusion

26, 37, 38, 41, 44, 69, 70

Group G — GWAS/QTL resources and large-scale mapping/context references

49, 50, 51, 60, 62

Group H — Interpretability / explainable AI (XAI) for genomics and plant phenotyping

53, 54, 56, 57, 58, 74

Group I — Reviews, benchmarks, and software/tooling for GP/AI in breeding (evaluation, pipelines, adoption)

12, 46, 52, 59, 61, 63, 64, 66, 75

Group J — Sustainability, energy/carbon cost, and orphan crop improvement

71, 72, 73

Group K — Stress biology and time-series omics/phenotyping (incl. hyperspectral ML)

76, 77, 78, 79, 80, 81
